# Targeting the hypoxic fraction of tumours using hypoxia-activated prodrugs

**DOI:** 10.1007/s00280-015-2920-7

**Published:** 2016-01-25

**Authors:** Roger M. Phillips

**Affiliations:** Department of Pharmacy, University of Huddersfield, Queensgate, Huddersfield, HD1 3DH UK

**Keywords:** Hypoxia-activated prodrugs, TH-302, AQ4N, EO9, Tirapazamine, PR-104, TH-4000, Hypoxia, Bioreductive drugs

## Abstract

The presence of a microenvironment within most tumours containing regions of low oxygen tension or hypoxia has profound biological and therapeutic implications. Tumour hypoxia is known to promote the development of an aggressive phenotype, resistance to both chemotherapy and radiotherapy and is strongly associated with poor clinical outcome. Paradoxically, it is recognised as a high-priority target and one of the therapeutic strategies designed to eradicate hypoxic cells in tumours is a group of compounds known collectively as hypoxia-activated prodrugs (HAPs) or bioreductive drugs. These drugs are inactive prodrugs that require enzymatic activation (typically by 1 or 2 electron oxidoreductases) to generate cytotoxic species with selectivity for hypoxic cells being determined by (1) the ability of oxygen to either reverse or inhibit the activation process and (2) the presence of elevated expression of oxidoreductases in tumours. The concepts underpinning HAP development were established over 40 years ago and have been refined over the years to produce a new generation of HAPs that are under preclinical and clinical development. The purpose of this article is to describe current progress in the development of HAPs focusing on the mechanisms of action, preclinical properties and clinical progress of leading examples.

## Introduction

**Fig. 1:**
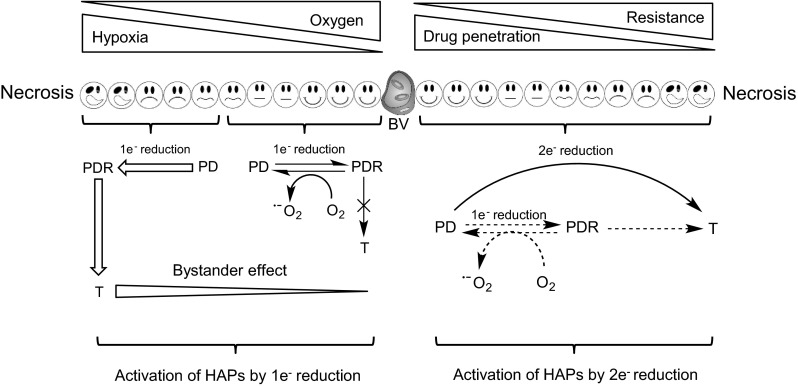
Cartoon of the hypoxic tumour microenvironment and a generalised scheme for the mechanistic activation of HAPs by one- and two-electron reductases under aerobic and hypoxic conditions. The cartoon describes a central blood vessel (BV) with tumour cells residing various distances away from the vascular supply. Cells that reside close to the blood vessel are ‘happy’ in that they are receiving nutrients and oxygen but as you move further away from the vessel, conditions become more stressful in terms of lack of oxygen (hypoxia) and nutrients (together with other physiological changes such as acidic extracellular pH) until conditions can no longer support cell viability and necrosis occurs. As distance from the supporting blood vessel increases, resistance to radiotherapy and chemotherapy increases and the delivery of drugs to hypoxic cells becomes increasingly problematical. The left-hand side of the cartoon describes the activation of HAPs by one-electron reduction pathways. The prodrug (PD) is reduced to a prodrug radical (PDR) which in the presence of oxygen redox cycles back to the parent compound generating superoxide radicals. In the absence of oxygen, the PDR is able to undergo further reactions (fragmentation or disproportionation) to generate the active toxic drug (T). Once the active drug has formed, it ideally should be able to diffuse back into the aerobic fraction and create a bystander effect. Even with a good bystander effect, HAPs are typically used in combination with radiotherapy or chemotherapy to eradicate the aerobic fraction. The right-hand side of the figure describes the activation of HAPs by two-electron reduction pathways. In this case, two-electron reduction bypasses the oxygen-sensitive PDR step leading directly or indirectly to the formation of the active toxic drug. This pathway is typically oxygen insensitive, and both the aerobic fraction and hypoxic fraction can theoretically be targeted. These pathways for HAP activation are generally applicable to most HAPs although exceptions do exist. AQ4N, for example, is reduced by sequential two-electron reduction steps that are inhibited by oxygen as described in the main body of the text

One of the characteristic features of solid tumour biology is the presence of a poor and inadequate blood supply [1]. This leads to the establishment of microenvironments that are characterised by gradients of oxygen tension, nutrients, extracellular pH, catabolites and reduced cell proliferation, all of which vary as a function of distance from a supporting blood vessel (Fig. 1). These microenvironments can be chronic in nature caused by poor blood supply (diffusion limited) or acute caused by the temporal opening and closing of blood vessels (perfusion limited). Hypoxia in tumours has been the focus of intense research for over 60 years, and both diffusion-limited hypoxia and perfusion-limited hypoxia are established features of solid tumours [2]. A third mechanism to explain the induction of hypoxia in tumours has been described, namely longitudinal arteriole gradients whereby oxygen-rich inflowing blood vessels branch and coalesce to form poorly oxygenated outflowing blood [3]. In this model, hypoxia would be formed along the axis of the vessel over a multimillimetre range, which contrasts with the submillimetre distances typically associated with perfusion- and diffusion-limited hypoxia. The origins of tumour hypoxia are therefore linked to the abnormal vascular supply that develops within tumours, and there is a substantial body of evidence demonstrating that hypoxia is a common feature of most if not all-solid tumours.

The presence of hypoxia in tumours has significant biological and therapeutic implications. Biologically, hypoxia is implicated in promoting resistance to apoptosis [4], suppression of DNA repair pathways and promotion of genomic instability [5] increased invasion and metastasis [6], promotion of angiogenesis [7], modulation of tyrosine kinase-mediated cell signalling pathways [8], evasion from immune surveillance [9], induction of autophagy [10], hypoxia-driven changes in central metabolic pathways [11], global changes in the metabolome [12], production of L-2-hydroxyglutarate leading to altered histone methylation [13], metabolic adaptation to hypoxia-induced reductive stress [14] and the provision of a niche where cancer stem cells reside [15]. The plethora of effects on tumour biology is mediated largely by hypoxia-inducible factors (HIF) [16] although HIF1-independent hypoxia responses have also been described [17]. In terms of therapeutic implications, the seminal work conducted by Gray in the 1950s [18] provided the first evidence that hypoxia is an underlying cause of resistance to radiotherapy. Since then, hypoxia has been strongly implicated in resistance to several cytotoxic chemotherapy drugs and targeted therapeutics [19, 20]. Multiple mechanisms contribute to hypoxia-induced drug resistance, but as pointed out by Wilson and Hay [21], the generalisation that hypoxia causes resistance to all cytotoxic drugs must be viewed with caution as some drugs are effective under hypoxic conditions. This note of caution should also be extended to include targeted therapeutics following the demonstration that some (such as dasatinib) are preferentially active against cell lines in vitro under hypoxic conditions [22].

Whilst the extent and severity of hypoxia in tumours varies between tumour types and within individual tumours, the combined biological and therapeutic implications of hypoxia have a significant bearing on prognosis. There is now an extensive body of evidence demonstrating that hypoxia can adversely affect clinical outcome [23, 24] and this makes hypoxia a high-priority therapeutic target. The importance of hypoxia as a target has been recognised for many years, but the translation of preclinical strategies designed to target hypoxic cells into mainstream clinical use has remained stubbornly difficult to achieve. Two main approaches are being used to eradicate hypoxic cells (1) the use of ‘bioreductive drugs’ or ‘hypoxia-activated prodrugs (HAPs)’ and (2) molecularly targeted drugs aimed at exploiting biochemical responses to hypoxia, particularly HIF pathways. The current status of HIF-targeted strategies is beyond the scope of this article which focuses on HAPs, their mechanism of action and recent progress in the preclinical and clinical evaluation of leading compounds in this class of drugs. This article also describes novel approaches where HAP-based approaches are being used to improve the selectivity of targeted therapeutics.

## Hypoxia-activated prodrugs (HAPs): general principles

The concept of hypoxia-activated prodrugs arose largely from the seminal work on quinone-based derivatives of Mitomycin C by Alan Sartorelli in the early 1970s [25]. Initially, these early studies focused primarily on enzyme-activated prodrugs in a process called bioreductive activation under aerobic conditions, but this concept was extended to include hypoxia following the demonstration that Mitomycin C preferentially killed hypoxic cells in vitro [26]. Over recent years, the term HAP has become established, but HAPs and bioreductive drugs are terms that are often used interchangeably. The principles underpinning the development of HAPs have been refined over the years, and the ‘ideal HAP’ should possess the following properties (1) ability to penetrate from a blood vessel to hypoxic cells within its pharmacokinetic lifespan; (2) preferential activation oxygen conditions that are low enough to prevent activation in normal tissues; (3) the reduced product should have the ability to kill non-proliferating cells typically found within the hypoxic fraction of tumours; and (4) the reduced product should have the ability to diffuse back into the proliferating aerobic fraction and exert a ‘bystander effect’ (Fig. 1).

Five different chemical entities have been shown to be capable of selectively targeting hypoxic cells, and these include nitro (hetero)-cyclic compounds, aromatic N-oxides, aliphatic N-oxides, quinone and transition metal complexes [27]. Whilst these are chemically distinct classes of compounds, a modular concept for the design of HAPs has been described with the three main components being (1) a trigger, (2) a linker and (3) an effector [28]. The effector is the cytotoxic component that is capable of killing cells within the hypoxic microenvironment, and these have typically been potent DNA-interactive agents. The purpose of the linker is to deactivate the effector, whilst the trigger group is the critical group that determines prodrug activation and hypoxia selectivity. Numerous trigger groups have been characterised, and these have to be enzymatically reduced (primarily by oxidoreductases) in order to release or activate the effector [28]. Both one- and two-electron oxidoreductases can catalyse the reduction of the prodrug, and selectivity is determined by the ability of oxygen to reverse the activation process and/or the overexpression of oxidoreductases in tumour tissue (Fig. 1). In general terms, one-electron reduction generates a prodrug radical species that can be back-oxidised in the presence of oxygen to generate the parental prodrug and reactive oxygen species. Host defence mechanisms can detoxify these radical species, thereby reducing toxic effects in oxygenated tissue, but in the absence of oxygen, the prodrug radical species undergoes further reduction/disproportionation or fragmentation reactions to generate products that are cytotoxic [21] Two-electron reduction in contrast bypasses the oxygen-sensitive prodrug radical intermediate, and activation of the prodrug is typically oxygen insensitive. In this case, selectivity is largely determined by the presence of elevated levels of enzyme in tumour tissue [29]. The mechanisms governing hypoxia-activated prodrug activation are summarised in Fig. 1, and important exceptions to these generalised mechanisms are identified here and in the main body of text below. It is also important to state that hypoxia-directed therapeutic agents are unlikely to demonstrate single-agent activity and should be used in combination with radiotherapy and/or chemotherapy that targets aerobic cells. Furthermore, in order to become effective components of combination therapies, HAPs need to be sufficiently safe with minimal side effects.

**Fig. 2:**
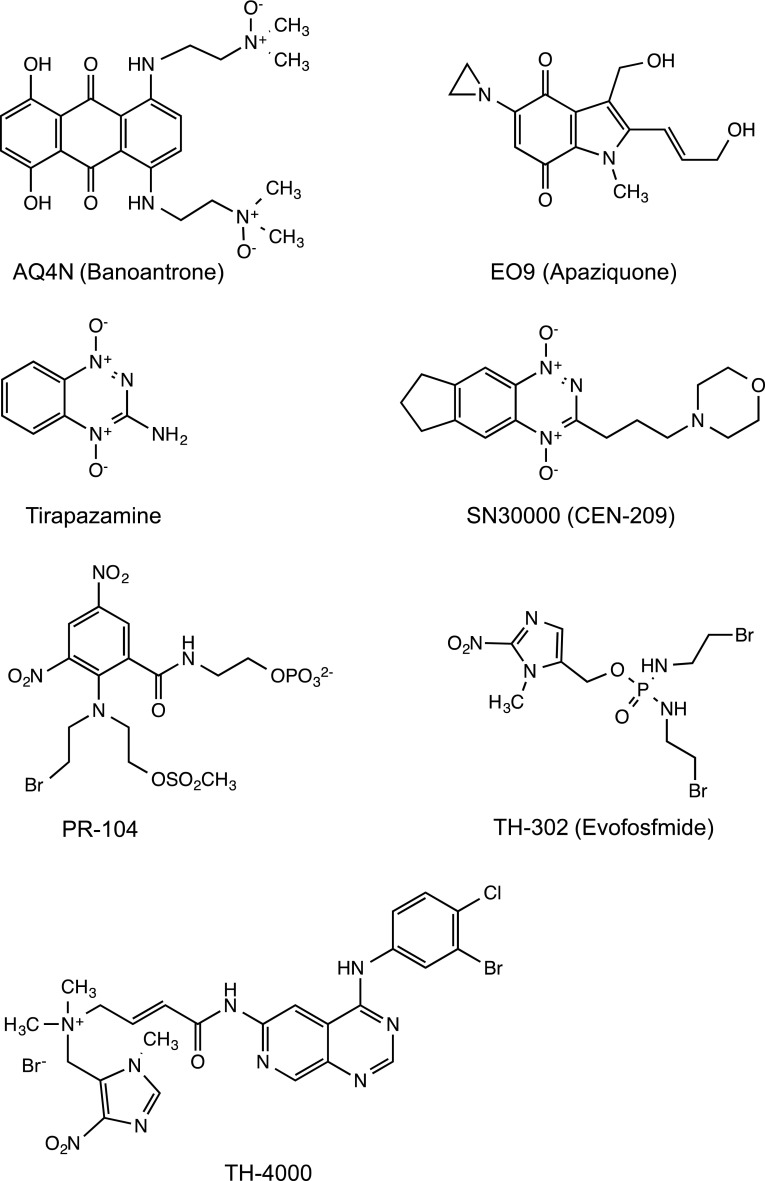
Structure of hypoxia-activated prodrugs/bioreductive drugs reviewed in this article. The chemical structures of other compounds that fall within this group of compounds can be found elsewhere [21, 27]

Whilst the concepts underpinning bioreductive drug and HAP activation are elegant and simple, success in this field has been very difficult to achieve and the stark reality is that despite over 40 years of pre-clinical and clinical research, no HAP has so far been approved for use in humans. Despite this lack of success, important lessons have been learnt from preclinical and clinical studies that have helped shape the design and evaluation of new HAPs. There is currently considerable optimism in the field, and in the following sections, recent progress in the pre-clinical and clinical evaluation of a series of selected HAPs (Fig. 2) is presented together with some emerging strategies for the next generation of HAPs.

### TH-302 (Evofosfamide)

**Fig. 3:**
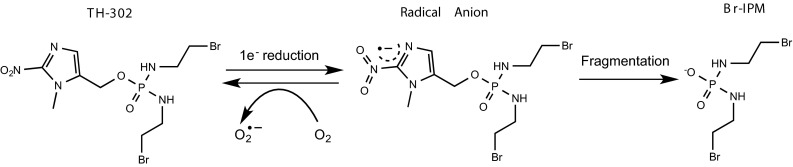
Reductive activation of TH-302 (Evofosfamide). In the presence of oxygen, the product of one-electron reduction (a radical anion) is rapidly converted back to the parent compound. At very low oxygen concentrations, the radical anion intermediate undergoes fragmentation to generate bromo-isophosphoramide mustard (Br-IPM) which is a potent alkylating agent

TH-302 is a 2-nitroimidazole HAP of bromo-isophosphoramide, the synthesis and initial preclinical evaluation of which was published in 2008 [30]. It was initially developed by Threshold Pharmaceuticals, and TH-302 is currently being developed in partnership with Merck. Its preclinical pharmacology has been described in detail elsewhere [31]. Its mechanism of action is presented in Fig. 3, and key features of its pharmacology include (1) reduction by one-electron oxidoreductases or radiolytic reduction leading to fragmentation and release of the potent DNA alkylating agent bromo-isophosphoramide mustard; (2) hypoxia-dependent induction of γH2AX, DNA cross-linking and cell cycle arrest; (3) broad activity against a range of cell lines with hypoxic cytotoxicity ratios (HCR) ranging from 11 to 600; (4) cells deficient in homology-dependent DNA repair, BRCA1, BRCA2 and FANCA exhibited marked sensitivity to TH-302 under hypoxia; (5) enhanced potency against H460 multicell spheroids compared to monolayer cultures; (6) exhibited a bystander effect in in vitro multicell layer models and in vivo; (7) broad anti-tumour activity across a panel of human tumour xenografts with clear evidence of selective eradication of hypoxic cells and neighbouring cells via the bystander effect; and (8) good pharmacokinetic and safety profiles in mice, rats, dogs and monkeys. More recent studies have confirmed that TH-302 targets hypoxic cells and potentiates the activity of doxorubicin and docetaxel in human tumour xenografts [32] and its activity in vivo can be enhanced by the induction of transient hypoxia [33]. As part of a combination regimen with cytotoxic drugs, targeted therapeutics or radiotherapy, TH302 has been shown to enhance anti-tumour activity and reduce the ability of hypoxic cells to repopulate tumours following re-oxygenation [34–40]. Activity is not confined to solid tumours but extends to acute myeloid leukaemia where TH-302 was effective against hypoxic cells that reside within the bone marrow microenvironment [41].

TH-302 is undergoing clinical trial, and phase I studies were published in 2011 [42, 43]. As a single agent administered intravenously, TH-302 was well tolerated. At maximum tolerated dose, adverse events included nausea, skin rash, fatigue and vomiting, and depending on the regimen used, dose-limiting toxicities were grade 3 skin and mucosal toxicities or grade 3 fatigue and vaginitis/proctitis [43]. Two partial responses in patients with metastatic small cell lung cancer and melanoma were observed with stable disease seen in 27 out of 57 patients [43]. A phase I study of TH-302 in combination with doxorubicin demonstrated that despite the haematologic toxicity of doxorubicin increasing when combined with TH-302, toxicities were manageable and partial responses were observed in 5 out of 15 patients with advanced soft tissue sarcoma [42]. Furthermore, one patient with advanced melanoma participating in the phase I studies had complete resolution of Cullen’s sign together with extracranial response in lung, liver and lymph node metastasis [44]. Promising clinical activity has also been reported in phase II trials involving TH-302 in combination with doxorubicin in advanced soft tissue sarcoma [45] and in combination with gemcitabine in patients with advanced pancreatic cancer [46]. TH-302 is undergoing further phase II trials against non-small cell lung cancer (with pemetrexed, NCT02093962) and advanced melanoma (NCT01864538), and phase III clinical trials against soft tissue sarcoma (NCT01440088) and pancreatic cancer (NCT01746979, MAESTRO study) are ongoing. The results of phase III studies are eagerly anticipated.

### EO9 (Apaziquone)

**Fig. 4:**
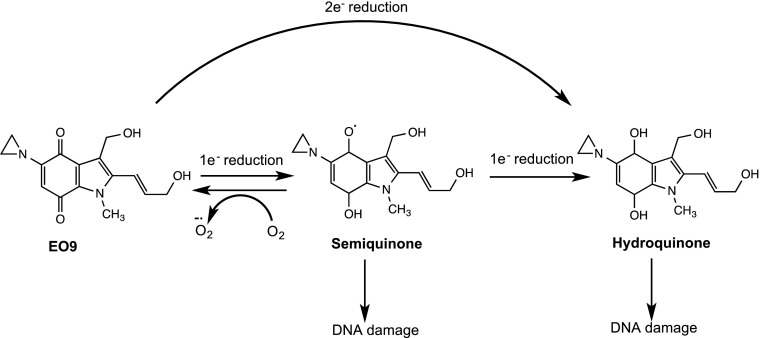
Reductive activation of EO9 (Apaziquone). One-electron reduction generates the semi-quinone radical which in the presence of oxygen redox cycles back to the parent compound. In the absence of oxygen, the free radical is stabilised or undergoes a further one-electron reduction to generate the hydroquinone species leading to DNA damage. This pathway predominates in cells that have low levels of the two-electron reductase NQO1, and very good selectivity for hypoxic cells can be achieved in cell lines that are devoid of NQO1. In NQO1-rich cells, two-electron reduction is the dominant route, and as this bypasses the oxygen-sensitive semi-quinone step, it is effectively an oxygen-insensitive route of activation. In this case, EO9 is equally active against aerobic and hypoxic cells and selectivity depends largely on the expression of NQO1 in tumour cells

EO9 is an indolequinone derivative of Mitomycin C that was originally synthesised in 1987 at the University of Amsterdam [47]. It has a chequered history, details of which have been reviewed recently [48]. Its mechanism of action (Fig. 4) involves reduction by one- and/or two-electron oxidoreductases to generate DNA-damaging species in both aerobic and hypoxic conditions. The two-electron reductase NAD(P)H:Quinone oxidoreductase 1 (NQO1 or DT-diaphorase) plays a central role in the bioreductive activation process and largely determines its ability to target aerobic or hypoxic cells [29]. EO9 has the ability to function as a classical HAP but only in cell lines that have low or no NQO1 [49, 50]. Under these conditions, one-electron reduction by enzymes such as cytochrome P450 reductase generates the semi-quinone radical which can redox cycle back to the parent compound in the presence of oxygen. HCR values in excess of 100 have been reported in a number of cell lines but particularly marked hypoxia selectivity occurs in cell lines that harbour the C609T single-nucleotide polymorphism that is devoid of NQO1 activity [49, 51]. In cell lines with high NQO1, however, the oxygen-insensitive two-electron reduction pathway dominates and HCR values close to 1 are typically reported [50]. In these cell lines, a good correlation between NQO1 activity and chemosensitivity under aerobic conditions exists [50]. Preclinical studies therefore demonstrated that EO9 has the ability to target both the aerobic fraction of NQO1-rich tumours (where selectivity is determined by elevated levels of NQO1 in tumours) and the hypoxic fraction of NQO1-deficient tumours [29].

Based on these and other favourable preclinical properties [52], EO9 was selected for clinical evaluation under the auspices of the New Drug Development Office in Amsterdam with phase I studies reporting partial responses in two patients with carcinomas of unknown origin and one in bile duct cancer [53, 54]. The results of phase II studies were, however, disappointing with no partial or complete responses observed. These studies concluded that EO9 had no clinical activity against NSCLC, pancreatic, breast, colorectal and gastric cancers [55, 56]. Several possible explanations were put forward to explain the poor results of these studies including the fact that EO9 was not evaluated as a classical HAP as it was only tested as a single agent. Furthermore, tumour enzymology and the presence of hypoxia in patient’s tumours were not incorporated into the design of the trials [57]. Whilst these issues represent important deficiencies in clinical trial design, it was argued that at least some patients would have had the right ‘biochemical machinery’ to activate EO9 and other explanations for why EO9 failed must exist.

Research focused on the issue of impaired drug delivery to tumours as a possible explanation. Whilst the factors that determine drug delivery to tumours are complex, systemic pharmacokinetic profiles and the ability to extravasate and penetrate through several layers of tumour cells to reach the hypoxic fraction are two key parameters [58]. Phase I studies had already established that EO9 was rapidly cleared from the systemic circulation following intravenous administration with half-lives of less than 20 min [54]. This combined with experimental evidence demonstrating that EO9 does not rapidly penetrate multicell layers in vitro suggested that EO9 will not penetrate more than a few cell layers from a blood vessel within its pharmacokinetic lifespan [59]. One method pursued to tackle this problem was to develop analogues of EO9 with better drug delivery properties, but an alternative approach designed to use EO9’s bad properties to our advantage was adopted. In the case of superficial transitional cell carcinoma of the bladder, chemotherapy is administered directly into the bladder by a catheter (intravesical administration). Intravesical administration of EO9 into the bladder would (1) circumvent the drug delivery problem observed following intravenous administration; (2) retention within the bladder for up to 1 h would extend the time EO9 was in contact with the tumour and enhance penetration; and (3) any drug that reached the systemic circulation would be rapidly cleared, thereby reducing the risk of systemic toxicity.

Following the demonstration that superficial transitional cell carcinoma of the bladder possessed the correct biochemical machinery required to activate EO9 [60], a new clinical trial was developed. Spectrum Pharmaceuticals sponsored the phase I study, and EO9 was administered directly into the bladder (intravesical administration) once a week for 6 weeks followed by assessment of anti-tumour efficacy 2 weeks after the final instillation. Prior to the administration of EO9, patients with multiple tumour lesions had all but one tumour surgically removed with the remaining tumour left to serve as a ‘marker lesion’ for assessing response. Complete response was defined as total ablation of the marker lesion, and eight complete responses were obtained out of a total of 12 patients entered into the study [61]. Using an identical trial design, similar complete response rates (30 out of 45 patients) were reported in phase II studies [62], and recurrence-free rates at 2 years were good in comparison with other marker lesion studies [63, 64]. Following the demonstration that a single intravesical administration of EO9 within 24 h of transurethral resection was well tolerated with a good safety profile [65], two phase III trials commenced using this new administration schedule (NCT00598806 and NCT00461591). In April 2012, Spectrum Pharmaceuticals announced that the results of these two trials did not meet their primary endpoint of a statistically significant difference in the rate of tumour recurrence at 2 years, but analysis of the pooled data from both studies showed a statistically significant effect in favour of EO9. A further phase III study using a multi-instillation schedule (once a week for 6 weeks) has been planned (NCT01410565).

### AQ4N (Banoxantrone)

**Fig. 5:**
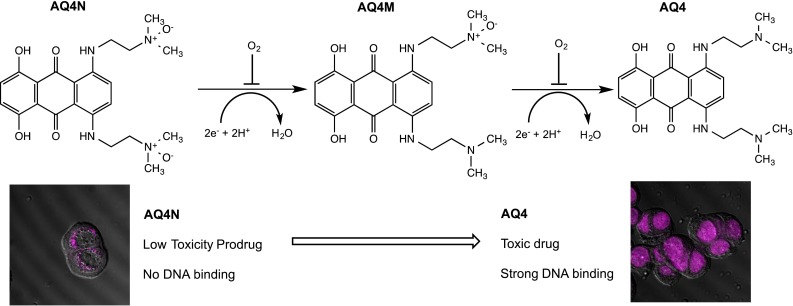
Reductive activation of AQ4N (banoxantrone). AQ4N undergoes sequential two-electron reduction by various cytochrome P450 (CYP) isoforms and inducible nitric oxide synthase to the mono-N-oxide (AQ4M) followed by a further two-electron reduction to AQ4. Oxygen inhibits the reduction process by outcompeting AQ4N for the haem centre of CYPs, and this mechanism differs fundamentally from other HAPs. The images are confocal images of cells treated with pure AQ4N and AQ4. No DNA binding is observed in cells treated with AQ4N, but fluorescence in the nuclei of AQ4-treated cells indicates strong DNA binding, leading to topoisomerase inhibition. The use of radiotherapy or chemotherapy to eradicate aerobic tumour cells causes re-oxygenation of hypoxic cells, and AQ4 prevents these cells repopulating the tumour by virtue of its strong inhibition of topoisomerases

AQ4N is an aliphatic N-oxide that is metabolised by cytochrome P450 (CYP) isozymes and inducible nitric oxide synthase (iNOS) to AQ4, a potent inhibitor of topoisomerase II [66, 67]. Its mechanism of action is summarised in Fig. 5 and involves an initial two-electron reduction step to the mono-N-oxide (AQ4M) followed by a further two-electron reduction to generate AQ4. Selectivity for hypoxic cells occurs because oxygen outcompetes AQ4N for the haem-centred active site of CYPs and oxygen therefore effectively inhibits the reduction in AQ4N [68]. Activation not only occurs in tumour cells but also in hypoxic tumour-associated macrophages where the induction of iNOS under hypoxic conditions led to reduction in AQ4N and killing of tumour cells via a bystander effect [69]. One of the key features of its mechanism of action is that AQ4 is a stable reduction product that strongly binds non-covalently to the DNA of hypoxic cells. Following the eradication of aerobic cells by radiotherapy and/or chemotherapy, hypoxic cells undergo a period of re-oxygenation and can repopulate the tumour. AQ4 inhibits the topoisomerase activity of hypoxic cells as they attempt to re-enter the cell cycle during these periods of re-oxygenation, and in preclinical models in vivo, AQ4N in combination with radiotherapy substantially enhances anti-tumour efficacy [70, 71]. Similar effects were seen with combinations of AQ4N and thiotepa, cyclophosphamide, cisplatin and the structurally related drug mitoxantrone [71–73]. More recent studies have demonstrated that the use of AQ4N following androgen deprivation therapy using bicalutamide (which induces hypoxia in tumours) significantly improves treatment outcome in prostate cancer xenografts [74]. Similarly, recent studies have also demonstrated that AQ4N has anti-metastatic properties by targeting hypoxic lesions in the lymph nodes and lymphatics in xenograft models [75]. With regard to drug delivery to the hypoxic fraction, studies in tumour models in vivo demonstrate that AQ4N has the ability to penetrate into hypoxic regions of tumour [73, 76]. Taken together, the preclinical profile of AQ4N clearly illustrates its potential as a HAP that is capable of tackling the key issue of tumour repopulation by hypoxic cells following the eradication of the aerobic fraction by conventional radio/chemotherapy. In addition to its HAP properties, AQ4N has also been shown to have anti-angiogenic properties under aerobic conditions, selectively targeting endothelial cells at low doses, inhibiting migration, tube formation, aortic ring vessel sprouting and invasion of proliferating endothelial cells [77]. Whilst the mechanism of action responsible for its anti-angiogenic properties is not fully understood (significant disruption of microtubule networks was observed), this mechanism could potentially contribute to the anti-tumour activity of AQ4N.

AQ4N has undergone clinical evaluation, and three phase I studies have been reported, two of which have evaluated AQ4N as a single agent [78, 79] and one in combination with radiotherapy [80]. As a single agent, AQ4N was well tolerated up to a maximum tolerated dose of 768 mg/m^2^ (administered intravenously as a 30-min infusion on days 1, 8 and 15 of a 28-day cycle) with the most common adverse events being fatigue, diarrhoea, nausea, vomiting, anorexia and blue discolouration of skin and body fluids [79]. The pharmacokinetic profile of AQ4N was dose dependent with low levels of AQ4M, and no AQ4 detected in the systemic circulation. Three patients had stable disease, two with bronchoalveolar lung cancer and ovarian cancer and the third with collecting duct renal cancer had prolonged stable disease for 25 months [79]. In a phase I proof-of-principle pharmacodynamics study, AQ4N at a dose of 200 mg/m^2^ (single dose administered intravenously using a 30-min infusion) was administered 12–36 h before multiple samples of tumour and normal tissue were surgically removed from each patient. AQ4N and its metabolites were analysed by LC/MS, the distribution of AQ4 relative to blood vessels determined by confocal microscopy and the relationship between AQ4 levels and the expression of the endogenous hypoxia marker Glut-1 determined by immunohistochemistry [78]. This study demonstrated that AQ4N was activated selectively in hypoxic regions of solid tumours and the levels of AQ4 detected exceeded those required for activity in animal models. In addition, high levels of AQ4 were detected in glioblastoma multiforme, indicating that AQ4N effectively crossed the blood–brain barrier [78]. In combination with radiotherapy, AQ4N was well tolerated up to 447 mg/m^2^ administered intravenously with no dose-limiting toxicity and tumour AQ4 concentrations also exceeded levels required for activity in preclinical models. Of the eighteen patients that were assessable for response, one had a partial response, two had >50 % tumour volume reduction and nine patients had stable disease [80]. Whether these responses were due to AQ4N or radiotherapy alone was not possible to determine but the results of this study illustrates the potential value of combination studies of AQ4N with radiotherapy. Regrettably, the clinical development of AQ4N has stalled, but new analogues of AQ4N are under development by OncoTherics.

### PR-104

**Fig. 6:**
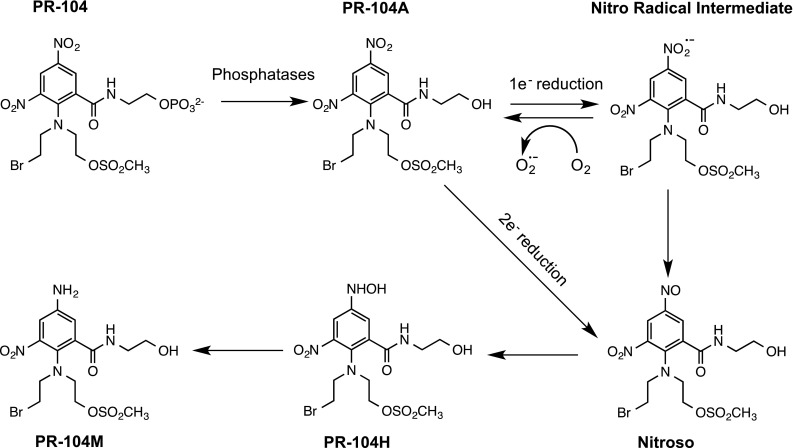
Reductive activation of PR104. The pre-prodrug PR-104 is converted to PR-104A by phosphatases in the systemic circulation, and this then undergoes either one- or 2-electron reduction. One-electron reduction by enzymes such as cytochrome P450 reductase generates a nitro radical intermediate that can undergo rapid redox cycling back to the parent compound in the presence of oxygen. In the absence of oxygen, the nitro radical intermediate undergoes a series of reactions leading to the formation of the toxic PR-104H and PR-104M derivatives. Two-electron reduction by aldo–ketoreductase 1C3 (AKR1C3) bypasses the oxygen-sensitive intermediate and generates the active metabolites under aerobic and hypoxic conditions. In this case, selectivity may be determined by the expression of AKR1C3 in tumour cells

PR-104 is a nitroaromatic compound that is a water-soluble phosphate ester ‘pre-prodrug’ of PR-104A, originally developed by the University of Auckland [81]. Its mechanism of action has been described in detail elsewhere [27] and is summarised in Fig. 6. Briefly, PR-104 undergoes rapid hydrolysis by systemic phosphatases to generate PR-104A that is metabolised by one- and/or two-electron oxidoreductases to the para-hydroxylamine PR-104H and para-amine PR-104M metabolites resulting in interstrand DNA cross-linking. Various oxidoreductases have been shown to catalyse the reduction in PR-104A including cytochrome P450 reductase [82], FAD-dependent oxidoreductase domain containing 2 (FOXRED2 [83]) and aldo–ketoreductase 1C3 (AKR1C3) [84]. PR-104 can target both hypoxic and aerobic cells with one-electron reduction pathways catalysed by cytochrome P450 reductase accounting for the majority of activity under hypoxia [85]. Reduction by AKR1C3 (oxygen-insensitive two-electron reduction process) accounts for activity under aerobic conditions [84]. Hypoxia selectivity in vitro is governed by the fact that the product of one-electron reduction undergoes redox cycling back to the parent compound and hypoxia together with reductase activity and repair of DNA interstrand cross-links are key variables that determine response to PR-104 [86]. More recent studies have also demonstrated that PR-104 is particularly active against hypoxic regions of triple-negative breast cancers that have dysfunctional homologous recombination repair pathways [87].

PR-104 has activity against a range of in vivo preclinical models, and its properties mean it can target hypoxic tumours and/or the aerobic fraction of tumours expressing AKR1C3. Against T cell acute lymphoblastic leukaemia xenografts, single-agent PR-104 treatment proved more efficacious compared to a combination of vincristine, dexamethasone and l-asparaginase and activity correlated with AKR1C3 expression [88]. This study also concludes that AKR1C3 expression could be used as a biomarker to select patients most likely to benefit from PR-104 treatment in future clinical trials. Similar studies reported complete responses in acute lymphoblastic leukaemia models and objective responses in other solid tumours, but in contrast, tumour response did not correlate with AKR1C3 levels [89]. Other studies have demonstrated that PR-104 could be used to eradicate acute lymphoblastic leukaemia cells residing in hypoxic niches in the bone marrow [90]. Against solid tumours, experimental and in silico modelling demonstrate that PR104/PR104A is able to penetrate into severely hypoxic regions of tumours where it is preferentially metabolised to cytotoxic metabolites [91]. A combination of experimental and modelling techniques have also demonstrated that PR-104A can exert a bystander effect that is predicted to contribute significantly to the anti-tumour efficacy of PR-104 [92]. PR-104 has demonstrated anti-tumour activity as a single agent against a range of solid tumour xenografts and greater than additive effects have been reported when PR-104 is used in combination with chemotherapy agents such as gemcitabine, docetaxel and sorafenib [81, 93] and/or radiotherapy [87]. The use of pharmacological approaches to induce tumour hypoxia has also been shown to potentiate the activity of PR-104 [94].

PR-104 is undergoing clinical trials, and several phase I clinical trials have been completed. As a single agent, a maximum tolerated dose of 1100 mg/m^2^ was reported following a one dose every 21-day schedule [95] and 675 mg/m^2^ when given on a one dose per week for 3-week schedule [96]. In both studies, PR-104 was administered by a 1-h intravenous infusion. Dose-limiting toxicities included fatigue, febrile neutropenia and infection following a once a week, every 21-day schedule and thrombocytopenia and to a lesser extent neutropenia using the more intensive schedule [95, 96]. No objective responses were reported in these studies despite the fact that PR-104A plasma AUC values exceeded the levels required for activity in preclinical models [95]. In combination with either gemcitabine or docetaxel, severe dose-limiting myelotoxicity occurred, the impact of which was reduced by prophylactic G-CSF in the case of docetaxel [97]. A combination of PR-104 and sorafenib in advanced hepatocellular carcinoma was also poorly tolerated with significant thrombocytopenia and neutropenia reported [98]. PR-104A undergoes glucuronidation [99], and it was suggested that reduced clearance due to compromised glucuronidation in patients with advanced hepatocellular carcinoma of PR-104A was partly responsible for the toxicity observed [98]. Recent studies in mice that do not significantly glucuronidate PR-104A confirm that the development of analogues of PR104 that are not readily glucuronidated may be able to exploit elevated AKR1C3 and/or hypoxia in hepatocellular carcinoma in humans [93]. Based on strong preclinical data, a phase I/II study in acute myeloid leukaemia (AML) and acute lymphoblastic leukaemia (ALL) has demonstrated clinical activity in 10 out of 31 patients with AML and 2 out of 10 patients with ALL [100]. PR-104 treatment also decreased the number of hypoxic cells in the bone marrow. These positive results indicate that PR-104 is able to exploit the hypoxic niche in acute leukaemias and further clinical evaluation in this setting is warranted.

### Tirapazamine (TPZ)

**Fig. 7:**
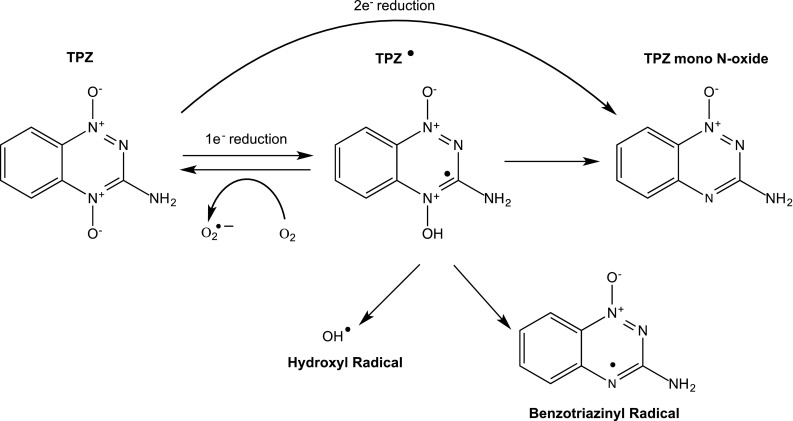
Reductive activation of Tirapazamine (TPZ). TPZ is reduced by both one- and two-electron reductases, but reduction by the former is considered to be the principle route of activation and selectivity for hypoxic cells. One-electron reduction generates a TPZ radical intermediate which undergoes redox cycling back to TPZ in the presence of oxygen. Under hypoxic conditions, the TPZ radical intermediate undergoes further reaction to produce hydroxyl and benzotriazinyl radicals that ultimately lead to the induction of DNA damage. Two-electron reduction by enzymes such as NQO1 generates metabolites with low activity and is regarded as a detoxification pathway

TPZ is an aromatic N-oxide that was originally developed in the mid-1980s, and its pharmacological properties have been extensively reviewed elsewhere [21]. Its mechanism of action is summarised in Fig. 7, and briefly, TPZ is reduced by one-electron reductases (such as cytochrome P450 reductase) to generate a radical species which in the absence of oxygen undergoes further spontaneous reactions, leading to the formation of DNA-damaging oxidising hydroxyl or benzotriazinyl radicals [101]. Selectivity for hypoxic cells is determined by the ability of oxygen to reverse the one-electron reduction step resulting in back oxidation to the parent compound and the formation of superoxide. The two-electron reductase NQO1 can also reduce TPZ, but this is considered a bioprotective mechanism as it bypasses the TPZ radical and forms the relatively non-toxic mono-N-oxide [102]. High hypoxia selectivity has been reported in a number of cell lines, and in vivo studies demonstrated that TPZ in combination with radiotherapy and cisplatin was highly effective against a range of human tumour xenografts [103] and TPZ entered clinical trial in early 1990s.

TPZ has been extensively evaluated in the clinic. Both phase I and II studies generated positive results, particularly phase II studies where TPZ was used in combination with cisplatin, etoposide and/or radiotherapy [104–107]. Unfortunately, several phase III clinical trials have failed to demonstrate any survival advantage by adding TPZ to chemotherapy or radiotherapy in non-small cell lung cancer [108], head and neck cancer [109] and cervical cancer [110]. Reasons for the failure of TPZ include failure of radiotherapy protocol compliance and lack of stratification of patients based on tumour hypoxia levels [111, 112]. Subsequent subgroup analysis of these trials using a range of endogenous markers of hypoxia proved of limited benefit in both head and neck cancers and NSCLC trials [113–115]. Whilst better methodologies for measuring hypoxia could have been employed [116, 117], the lack of a correlation between clinical response and hypoxia markers supports the clinical findings that inclusion of TPZ into combination protocols has limited if any clinical benefit. An alternative explanation for the failure of TPZ is relatively poor drug penetration into hypoxic regions of tumours. Because TPZ can be activated under comparatively mild hypoxia, it has been shown that TPZ is metabolised too rapidly to penetrate deeply into severely hypoxic tissue [118]. Using a combination of in silico models and experimental approaches, analogues of TPZ that have better penetration and metabolism properties have been developed with SN30000 (now known as CEN-209 following licensing to Centella) emerging as a candidate for clinical development [119]. Details of its mechanism of action have been described elsewhere [120], and SN30000 is likely to proceed to phase I clinical trials shortly. It is hoped that the valuable experience gained from the TPZ clinical trials is incorporated into the design of these trials [111].

### TH-4000

**Fig. 8:**
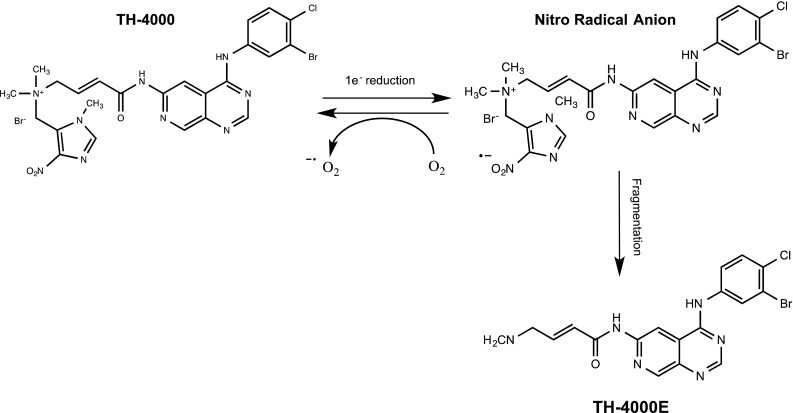
Reductive activation of TH-4000. Reduction in TH-4000 leads to fragmentation of the prodrug and release of a potent inhibitor of EGFR (TH-4000E). Selectivity for hypoxic cells is determined by redox cycling of the nitro radical anion species in the presence of oxygen

The vast majority of HAPs developed to date result in the generation of metabolites that damage DNA either directly or indirectly. TH-4000 represents a significant departure from this mechanism and is one of the first hypoxia-activated molecularly targeted therapeutic to be developed. Discovered at the University of Auckland and initially referred to as PR-610 or Hypoxin™, TH-4000 is a hypoxia-activated EGFR tyrosine kinase inhibitor (TKI) that is now being developed by Threshold Pharmaceuticals. It is designed to release an irreversible EGFR TKI within hypoxic regions of tumours (Fig. 8), thereby improving selectivity and circumventing some of the toxicities observed with existing EGFR TKIs. Preclinical studies have been published in abstract form [121, 122] where TH-4000 was shown to be more active against NSCLC xenografts with wild-type and mutant EGFR than Erlotinib. TH-4000 is currently undergoing phase II clinical evaluation against EGFR-mutant, T790M-negative patients with advanced NSCLC (NCT02454842) and metastatic squamous cell carcinoma of the head and neck or skin (NCT02449681).

## Novel HAPs in preclinical development

**Fig. 9:**
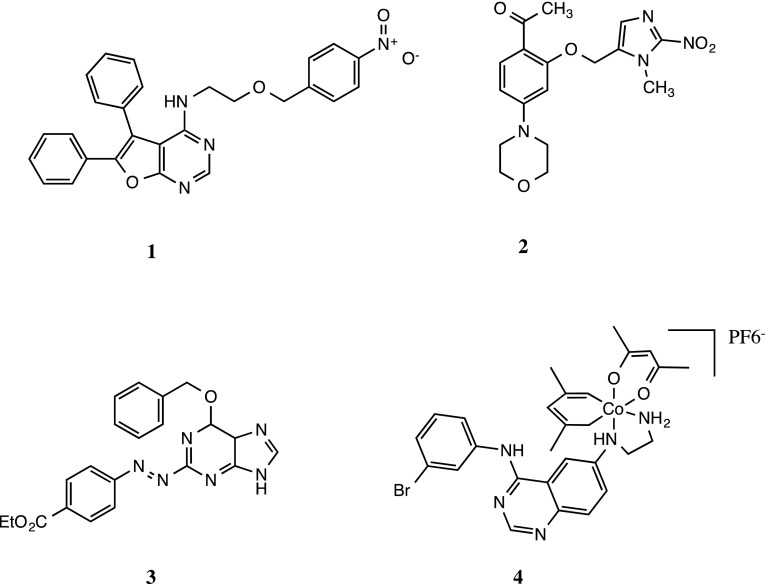
Chemical structures of novel HAPs currently under development. Compounds 1 to 4 include hypoxia-activated inhibitors of Chk1/Aurora A (CH-01), DNA-PK (BCCA621C), O^6^ alkylguanine DNA alkyltransferase and EGFR, respectively. Details of their preclinical pharmacology are described elsewhere [123–126]

Whilst HAPs designed to release DNA-damaging species are still being developed, other HAPs have been designed to release inhibitors of DNA damage response pathways [123, 124]. Release of the Chk1/Aurora A inhibitor CH-01 (Fig. 9) following reduction of a 4-nitrobenzyl hypoxia trigger leads to fragmentation and release of active Chk1/Aurora A inhibitor with potent activity against severely hypoxic cells in vitro [123]. Similarly, attachment of a DNA-PK inhibitor to a nitroimidazole hypoxia trigger group led to inactivation of the complex (BCCA621C, Fig. 9) and no cytotoxic activity under aerobic conditions was reported [124]. Under severe hypoxia, however, the inhibitor was released and was able to radiosensitise hypoxic H460 cells in vitro with a sensitiser enhancement ratio of 1.85. HAPs targeting the O^6^-alkylguanine DNA alkyltransferase (AGT, Fig. 9) pathway have also been developed and have shown hypoxia selective activity in a range of cell lines in vitro [125]. This approach could lead to the selective depletion of AGT in tumour tissue without corresponding depletion in normal tissue leading to sensitisation of tumours to O^6^ guanine targeting cytotoxic drugs such as temozolomide. Using a similar concept to TH-4000, a HAP strategy has been developed to release EGFR inhibitors using Cobalt (III) as the hypoxia-sensitive trigger group (Fig. 9) [126]. Hypoxia selective inhibition of EGFR was demonstrated in vitro, and anticancer activity in vivo against A431 and Calu3 human tumour xenografts was demonstrated. HAP strategies are not only being used to deliver targeted small molecule but have recently been extended to include siRNA approaches. Azobenzene hypoxia trigger groups have been linked to siRNA for hypoxia-targeted delivery of siRNA along with PEGylated nanopreparations in a proof-of-concept study. Using multicell spheroids, the construct was shown to penetrate into the spheroid mass, and in HeLa cells engineered to stably express GFP, a 30–40 % down-regulation of GFP was detected only under hypoxic (0.5 % oxygen) conditions [127].

## Concluding remarks and future directions

One of the most pressing unmet clinical needs is the development of therapeutic agents that can eradicate the hypoxic fraction of tumour cells. Despite extensive efforts to target and kill hypoxic cells over several decades, the need for such therapeutic strategies remains a significant objective. In the field of HAPs, several compounds have made it through pre-clinical development into clinical trial, but success has so far proved elusive. These failures, however, have helped shape the development and testing of new HAPs, and there is now genuine optimism that success is imminent. Of the compounds undergoing clinical development, TH-302 is currently the ‘gold standard’ and the results of phase III trials are eagerly awaited. As described in this article, there are a number of other HAPs in clinical trial and behind these, there is a pipeline of other agents undergoing preclinical evaluation or awaiting clinical trial. Of particular interest is the development of HAP strategies designed to release targeted therapeutics (pioneered by TH-4000) within the hypoxic microenvironment of tumours, and this is an exciting development. It should also be noted that HAPs represent one approach to targeting tumour hypoxia and other areas are being actively pursued [128]. One such avenue is tumour metabolism, and as the biology underpinning the metabolic phenotype of tumour cells and the metabolic interplay between tumour and host cells under hypoxia are unravelled, novel therapeutic targets and strategies will emerge. Despite its lack of immediate success, the field of HAP development has produced a wealth of knowledge, understanding and expertise. It is hoped that the novel approaches to targeting hypoxia under development now will take note of the principles and experience gained from over 40 years of developing HAPs and incorporate them into the design of appropriate preclinical and clinical studies.
